# Complications Related to Sexualized Drug Use: What Can We Learn From Literature?

**DOI:** 10.3389/fnins.2020.548704

**Published:** 2020-11-27

**Authors:** Hélène Donnadieu-Rigole, Hélène Peyrière, Amine Benyamina, Laurent Karila

**Affiliations:** ^1^Addictions Department, Saint Eloi Hospital, University Hospital of Montpellier, Montpellier, France; ^2^INSERM U 1058, Pathogenesis and Control of Chronic Infections (PCCI), Montpellier, France; ^3^Addictovigilance Center, Department of Medical Pharmacology and Toxicology, University Hospital of Montpellier, Montpellier, France; ^4^Centre d’Enseignement, de Recherche et de Traitement des Addictions, Hôpital Universitaire Paul-Brousse (APHP), Villejuif, France; ^5^Paris-Saclay University, Saint-Aubin, France; ^6^Unité de Recherche PSYCOMADD, Villejuif, France

**Keywords:** psychoactive substances, sexual behaviors, cathinones, sexualized drug use, GBL/GHB

## Abstract

Chemsex is described as the use of specific psychoactive substances (PS) during sexual activity to sustain, enhance, disinhibit or facilitate the sexual experience. It preferentially concerns men who have sex with men (MSM). They use new synthetic substances like cathinones, methamphetamines, gamma-butyrolactone/gamma-hydroxybutyrate (GBL/GHB), ketamine, and cocaine. The prevalence of chemsex varies from 3 to 31% during lifetime. The Internet has participated significantly in the evolution of sexual behaviors, both in terms of sexual dating and the availability of new synthetic substances. The advent of geolocation applications contributed to the development of chemsex. The literature describes many complications linked to these sexual practices; the main clinical effects related to cathinones consumption were psychiatric symptoms; agitation, hallucinations, anxiety, suicidal ideation, paranoia, and confusion. Regular GBL/GHB consumption alter cognitive functions, particularly memory and emotion management. Use of these drugs in party and play is dramatically associated with high-risk sexual behaviors. The prevalence of hepatitis B, hepatitis C syphilis, and HIV is higher in men who use methamphetamine and Viagra and/or who declared they practiced slamming, chemsex, and fisting. Other sexually transmitted infections (STIs) such as gonorrhea have increased with methamphetamine and GHB/GBL use. Actually, the care of individuals who practice Chemsex in a problematic way is currently not codified, but the use of integrative and specific interventions is necessary.

## Introduction

Drug use in a sexual context has been described since antiquity in order to improve sexual performance or to promote desinhibition. In this review we specifically studied the recent phenomenon of “chemsex” secondary to geolocation applications and new designer substances, which has increased exponentially since the 2000s. Numerous substances are linked to sex use and they are associated with different populations and sexual behaviors. Alcohol, cannabis and MDMA (methylene-dioxyméthamphétamine) are more commonly used in heterosexual practices (Lawn et al., 2019), whereas men who have sex with men (MSM) use cathinones, methamphetamines, gamma-butyrolactone/gamma-hydroxybutyrate (GBL/GHB), ketamine, and cocaine. This behavior is referred to “chemsex” (formerly “sexualized drug use”). Chemsex is described as the use of specific psychoactive substances (PS) during sexual activity to sustain, enhance, disinhibit, or facilitate the sexual experience. (Giorgetti et al., 2017). The Internet has participated significantly in the evolution of sexual behaviors, both in terms of sexual dating and the availability of new synthetic substances. Chemsex is associated with a high risk of contraction of sexually transmitted infection (STI) and bloodborne viruses (BBV). The care of individuals who practice Chemsex in a problematic way is currently not codified. The objective of this review was to describe complications related to drug use in a sexual context in order to adapt specific care.

## Methods

A review of the literature was conducted using PubMed. The search was carried out per theme using the keywords reported in Table 1.

**TABLE 1:** Keywords (from 2000 to 2020).

**Themes**	**Keywords**	**Alone/Association**
Sexual behavior	Chemsex Slamming	228 52/9
Drugs	Cathinones Mephedrone Methamphetamine GHB GBL GBL/GHB Ketamine Erectile designer drugs (Sildenafil, Tadalafil, and Vardenafil)	766/17 830/77 335/123 1704/34 456/23 10/3 13 711/76 2859/14
Applications	Sex meeting application	122/22

**FIGURE 1:**
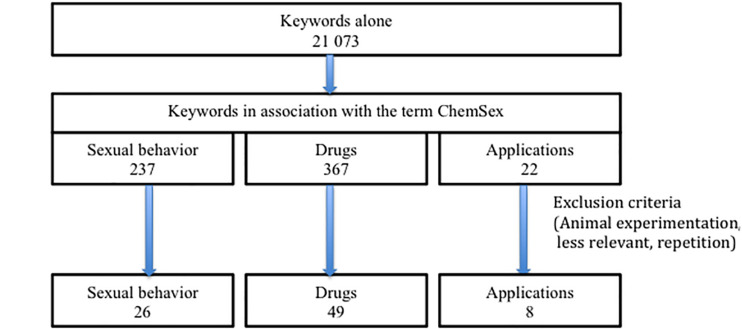
Flow chart.

These keywords were used alone or in association with the keyword “chemsex.” The Table 1 shows the number of articles identified for each keyword and in association from 2000 to 2020. This technique has allowed to find many articles with keyword alone and/or association with the main keyword “chemsex.” We have selected those that seemed more relevant for this review about complications related to drug use (Figure 1). Original animal articles have been excluded.

## Prevalence

Currently, the prevalence of chemsex is difficult to estimate because its definition varies between countries (Schmidt et al., 2016). It varies from 3 to 31% during lifetime and from 0.4 to 16.3% the last month. The frequency of chemsex depending on the city of residence and countries studied, and the taking into account or not of HIV-positive individuals (Elliot et al., 2017), the use of dating applications and the type of PS used (Maxwell et al., 2019).

Slamming is the term used for intravenous injection of these PS during party and play. In Australia, the prevalence of slamming is 10% in MSM, and in England it was found to be 16% in an MSM population diagnosed with *Shigella flexneri* 3a infection (Gilbart et al., 2015). Slamming is more common in MSM and women who have sex with women (WSW) than in libertine heterosexuals practicing chemsex (Heinsbroek et al., 2018). These differences in prevalence show how interesting it would be to define the practice of chemsex in an international way: target population, drugs used, and exclusion criteria.

## Sex Meeting Applications

The practice of combining sex and PS has increased steadily with the development of mobile applications. The advent of geolocation applications in 1990 transformed the way gay and bisexual men meet (Grov et al., 2014) and contributed to the development of chemsex. These new applications allow to meet one or more partners very quickly in their surroundings and to express their sexual desires, and their preferences in the practice of chemsex. Sex meeting applications made it possible for “clubbers” and “sexers” to meet. These 2 populations did not have the same sexual practices. “Sexers” had harder practices and used drugs, which have since become widespread amongst MSM.

At least 40% of MSM and 68% of the youngest individuals (Garofalo et al., 2007) find their partners using the Internet. Finding a sex partner on Internet or using a mobile application increases condomless anal intercourse and the risk of HIV transmission (Lewnard and Berrang-Ford, 2014; Whitfield et al., 2017). Sociologists are worried about the disappearance of non-virtual meeting places associated with the development of phone applications and describe the increase of solitude felt by homosexuals (Renninger, 2018).

## Designer Substances and Erectile Disorder Drugs (EDDs)

**FIGURE 2:**
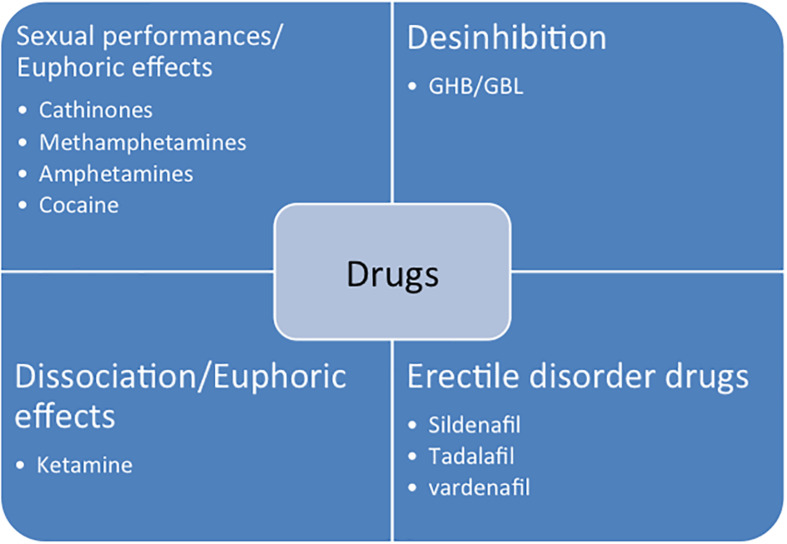
New designer drugs and their expected effects.

New designer substances appeared in the early 2000s and participated in the development of chemsex. New synthetic substances are detailed in the Figure 2 and in the Table 2. The main complications are described in Table 3. Most of these studies come from emergency or intensive care.

**TABLE 2:** New designer drugs and their expected effects.

**Drugs name**	**Common names**	**Expected effects**	**Way of administration**
Cathinones	MDPV 4-MMC Mephedrone 4-MEC M-Cath	Psychostimulants amphetamine-like effect Euphoria Empathy Increase libido and sexual performance	Orally Sniff Intrarectally (booty bump) Injection
Methamphetamine	Meth Crystal Ice	Powerful psychostimulant Euphoria Empathy	Orally Sniff Smoke Injection
GBL/GHB	“G”	Relaxation disinhibiting Increases desire facilitates penetration	Orally
Ketamine	“Ke”	Psychostimulant effect Euphoria	Sniff Orally

**TABLE 3:** Drugs and their main complications (without STI and BBV).

**Drugs**	**Main risks**	**Treatment**	**Studies**	**Number of subjects**
Cathinones	Psychiatric: panic attack, feeling of persecution, depression, confusion, hallucinations, suicide attempt or suicidal ideation, severe agitation, or violence	Psychiatric hospitalization Symptomatic treatment	Batisse et al., 2014 Spiller et al., 2011 Sande, 2016 Schmoll et al., 2018 Diestelmann et al., 2018	21 subjects 236 subjects 249 subjects 96 subjects 50 subjects
	Cardiac: hypertension, tachycardia, and chest pain Cardiac ischemia	Symptomatic treatment	Ezaki et al., 2016 Spiller et al., 2011	61 autopsy cases 236 subjects
	Dependence		Winstock et al., 2011	100 subjects
	Sympathomimetic syndrome and rhabdomyolysis	Intensive care Benzodiazepines Psychiatric care	O’Connor et al., 2015 Froberg et al., 2015 Umebachi et al., 2016	102 subjects 23 subjects 8 subjects
Methamphetamine	Cardiac: methamphetamine-associated cardiomyopathy, heart rhythm disturbance, acute coronary syndromes, pulmonary arterial hypertension, and hypertension	β-blockers Blockage of the renin–angiotensin system Aripiprazole	Sevak et al., 2011 Neeki et al., 2018	6 subjects 449 subjects
	Psychiatric: depression, confusion, acute psychosis and psychotic symptoms, and anxiety disorder	Antipsychotic medications Benzodiazepines	McKetin et al., 2013	278 subjects
	Neuropsychological effects: alteration of executive functions, episodic memory, psychomotor functions, and complex information processing speed.		Scott et al., 2007	Meta-analysis (18 studies)
	Dependence	*N*-acetyl-cysteine	McKetin et al., 2019	180 subjects
	Dental and periodontal disease		Shetty et al., 2015 Rommel et al., 2016	571 subjects 100 subjects
GBL/GHB	Coma, presentation to a emergency department, and neurological complications	Intensive care	Wood et al., 2013 Raposo Pereira et al., 2020 Raposo Pereira et al., 2018	158 subjects 27 subjects 27 subjects
	Dependence	Baclofen Diazepam	Bell and Collins, 2011	19 subjects

### Synthetic Cathinones

The most used PS are synthetic cathinones, the leader of which being mephedrone or 4 MMC (4-methylmetcathinone). Their development was inspired by the Khat plant (*Catha edulis*) that is considered as a natural amphetamine and is used in certain countries of East Africa and the Arabian Peninsula (Kalix, 1990). These new designer substances are sold as bath salts “not for human consumption” to circumvent the legislation (Coppola and Mondola, 2012). They belong to the phenylethylamine family (Petit et al., 2013) and are psychostimulants with an amphetamine- or cocaine-like effect. They generate agitation, euphoria, and empathy in users and increase libido and sexual performance.

These PS have a strong addictogenic effect with dependence levels of 30% according to DSM-IV criteria (Dargan et al., 2011; Winstock et al., 2011). The main acute complications are cardiac, psychiatric, and neurological, and they can be fatal (Busardo et al., 2015; Ezaki et al., 2016; Sande, 2016; Kronstrand et al., 2018; Riley et al., 2019; Table 3).

Blood levels in case of lethal intoxication are higher than in cases of non-lethal poisoning, and lethal situations arise from the combining of several drugs (Papaseit et al., 2017). These substances have been classified as narcotics in Europe since 2010 (Forsyth, 2012) and it is currently illegal to purchase or possess them, but they are usually obtained on the Internet or directly during party and play. Cathinones take the form of a white powder or small crystals. They are taken orally, by snorting, intrarectally (booty bump), or by injection (slamming).

In the study of Spiller et al. (2011) the main clinical effects (>10%) related to cathinones consumption were agitation (82%), combative violent behavior (57%), tachycardia (56%), hallucinations (40%), paranoia (36%), confusion (34%), myoclonus (19%), hypertension (17%), chest pain (17%), and mydriasis (13%).

In another serie, main complications of cathinones related to chemsex were psychotic symptoms, agitation, anxiety, suicidal ideation, or suicide attempt (Batisse et al., 2014). More recently, severe psychiatric symptoms have been observed with ephylone, a recent available synthetic cathinones, in a context of chemsex (Serre et al., 2019).

Excited delirium has been reported with the use of synthetic cathinones, with a challenging combination of paranoia, confusion, severe agitation, and violent behavior (Diestelmann et al., 2018; Schmoll et al., 2018). The presentation of these patients has been frequently complicated by evidence of skeletal muscle damage, dehydration, renal dysfunction, and hyperthermia that may lead to multiorgan failure and death. The precise pathophysiology underlying the syndrome of excited delirium is incompletely understood. However, the role of the central dopamine dysregulation, inducing a thermoregulation dysfunction has been suggested (Penders et al., 2012).

Cathinones are often combined with other illicit PS such as methamphetamine (Maxwell et al., 2019), GBL/GHB (Bourne et al., 2015; Edmundson et al., 2018), ketamine, and cocaine (Melendez-Torres et al., 2018b).

### Methamphetamine

Methamphetamine (crystal meth), like cocaine and amphetamines, inhibits the reuptake of monoamine transporters and stimulate the release of dopamine, noradrenaline, adrenaline, and serotonin (Liechti, 2015). Crystal is characterized by its stronger psychostimulant effects and its immense addictogenic potential. Use of this dangerous substance is mainly described in the United States and the United Kingdom (Degenhardt et al., 2010) with prevalence ranging from 27% (lifetime use) to 7% (recent use) (Benotsch et al., 2012). Methamphetamine can be swallowed, smoked injected or intrarectally administrated. Methamphetamine has been linked to a lot of cardiac, psychiatric, neuropsychological and dental effects (Table 3).

### Gamma-hydroxybutyrate/Gamma-butyrolactone

Gammahydroxybutyrate is a central nervous system depressant that has a double stimulant and sedative effect (Raposo Pereira et al., 2019b). GBL, which is cheaper, is taken orally in liquid form and transformed into GHB (Busardo et al., 2018).

Gamma-butyrolactone/gamma-hydroxybutyrate is most often mixed with another drink. The sought-after effects are relaxation, disinhibition, increased desire and sensuality, and easer penetration. The main acute risk is overdose with significant sleepiness and hypothermia that can lead to coma and death.

An increase in the number of deaths in London related to GHB use was observed between 2011 and 2015 with 61 reported deaths, while a 119% increase in deaths between 2014 and 2015 was observed versus 25% for cocaine (Hockenhull et al., 2017). Regular GBL/GHB consumption and repeated comas alter cognitive functions, particularly memory (Raposo Pereira et al., 2018) and emotion management (Raposo Pereira et al., 2019a; Table 3). GBL/GHB dependence can be established using physical and psychological criteria with consumption outside of sexual intercourse.

### Ketamine, Cocaine, and Speed

Ketamine, an anesthetic used in human or veterinary medicine, is a phencyclidine (PCP) derivative that blocks non-competitively the glutamate *N*-methyl-*D*-aspartate (NMDA) receptor (Corazza et al., 2013). It is used as a psychoactive substance in sexual sessions for its euphoric effects. It can cause hallucinations at higher doses. Urologic complications are described with ketamine. Cases of bladder dysfunction have been reported in the literature, mainly ulcerative cystitis. Cases of hydronephrosis have also been reported. Symptoms described by users are frequency and urgency of urination, dysuria, urge incontinence, and occasionally painful hematuria (Morgan and Curran, 2012).

Cocaine and speed are still used in a sexual context but less frequently since the advent of the new designer drugs (Busardo et al., 2019).

### Erectile Dysfunction Agents

Non-psychoactive substances such as erectile dysfunction agents (sildenafil, tadalafil, and vardenafil) are used to facilitate or enhance sexual performances. These drugs are diverted from their medical use to counterbalance the negative effects of psychoactive drugs and to prolong the duration of sexual intercourse (Giorgetti et al., 2017). Combining all these drugs is associated with high cardiac toxicity (Bracchi et al., 2015).

## STI and BBV

In addition to the complications already described, the major risks are related to the sexual behaviors of the users, and the injection of drugs that increases the risk of spreading BBV and STI.

Use of illicit and licit drugs in party and play is dramatically associated with high-risk sexual behaviors. Use of methamphetamine is strongly associated with condomless anal intercourses (Fisher et al., 2011), multiple sex partners (Melendez-Torres et al., 2018a), sex marathons, and sex with HIV-positive MSM (Benotsch et al., 2012; Bui et al., 2018). The prevalence of hepatitis B, syphilis (Rana et al., 2019), and HIV is higher in men who use methamphetamine and Viagra which is associated with serodiscordant unprotected sexual intercourse (Spindler et al., 2007). Hepatitis C seroprevalence is higher in HIV-positive MSM who declared they practiced slamming, chemsex, and fisting (Vaux et al., 2019). Other STIs such as gonorrhea have increased with methamphetamine and GHB/GBL use (Kohli et al., 2019) and there has been a worldwide increase in all STIs among MSM that is linked to chemsex and Internet, which facilitates high-risk sexual behaviors (Soriano and Romero, 2018).

## Prevention and Intervention

Phone applications would be ideal to promote messages in terms of sexual health, STI and prevention of addictions. Some applications have started to emit HIV prevention messages (Chan et al., 2016). Medina et al. (2019) explained that future HIV prevention approach should pass through dating applications. Moreover another study showed that interventions that facilitate condom negotiation could exist in future applications (Tang et al., 2016).

Providing care to individuals suffering from the problematic practice of chemsex is complex; there are no explicit recommendations on specific drugs that could be used for withdrawal or regulation and the care must simultaneously take into account pharmacological, addictological, and psychological and sexual aspects. The use of integrative and specific interventions is necessary. Different types of therapy are tested as cognitive behavior therapy, contingency management, and gay-specific cognitive behavior therapy (GCBT). These therapies are used with or without medications for withdrawal or maintaining abstinence. GCBT integrated elements from standard cognitive behavior therapy with cultural and social elements of chemsex users. Reback has proved the efficiency of this therapy in methamphetamines users in the United States (Reback and Shoptaw, 2014). CBT and motivation interview improve adherence to HIV medication in gay and bisexual men (Parson et al., 2018).

For GBL/GHB withdrawal, the most used drugs are benzodiazepines, neuroleptics, and sometimes barbiturates (Cappetta and Murnion, 2019). Serious complications may occur with these, including hallucinations, delusions, and epileptic fits (Liao et al., 2018; Neu, 2019). Baclofen has been tested for the long-term maintenance of GBL/GHB abstinence, but randomized studies are required before specific recommendations can be issued (Beurmanjer et al., 2018).

Drugs such as atypical antidepressants (mirtazapine) or naltrexone have been tested to reduce sexual risk-taking and metamphetamine use (Knight et al., 2019).

Fighting against the transmission of infections requires preventive interventions and harm reduction including condom distribution and needle exchange programs. These practices need to be developed.

Special attention should be paid to MSM with problematic chemsex behavior so they may have access to the best possible post-exposure prophylaxis (PEP) and pre-exposure prophylaxis (PrEP) (Hammoud et al., 2018; Sewell et al., 2019).

Actions focusing on prevention, addiction management and sexual health need to be increased and new community spaces such as sexual health centers need to be opened to break down barriers and help alleviate the shame of chemsex drug users (Giorgetti et al., 2017; Sewell et al., 2018).

## Conclusion

Chemsex is a complex issue. The behavior is at the crossroads of sociology, infectious medicine, addiction, and sexology. Its users are confronted with medical, psychiatric, and sexual risks. Some users engage in chemsex seeking sexual disinhibition, others in order to embrace their sexual preferences, and others still for the effects of the drugs.

The phenomenon can be a claim of part of the MSM population that wants to have unbridled sex with protection against HIV. There is still enormous stigma and shame associated with HIV infection and being homosexual (Dubov et al., 2018). With chemsex, caregivers are confronted with a continuum between normal and pathological sexual and drug use behaviors. The care to be provided to chemsex users must be validated by large sample studies. CGBT and LGBT-specific-e-therapy (Lucassen et al., 2018) are interesting ways to facilitate prevention and access to care for problematic chemsex users.

## Author Contributions

HD-R and HP reviewed the literature and wrote the manuscript. LK and AB revised the manuscript. All authors contributed equally to this manuscript.

## Conflict of Interest

The authors declare that the research was conducted in the absence of any commercial or financial relationships that could be construed as a potential conflict of interest.

## Abbreviations

PS: psychoactive substances
BBV: bloodborne viruses
STI: sexually transmitted infections
MSM: men who have sex with men
EDDs: erectile disorder drugs.

## References

[B1] Batisse A. Fortias M. Bourgogne E. Grégoire M. Sec I. Djezzar S. (2014). Case series of 21 synthetic cathinones abuse. *J. Clin. Psychopharmacol.* 34 411–413. 10.1097/jcp.0000000000000116 24743721

[B2] Bell J. Collins R. (2011). Gamma-butyrolactone (GBL) dependence and withdrawal. *Addiction* 106 442–447. 10.1111/j.1360-0443.2010.03145.x 20925687

[B3] Benotsch E. G. Lance S. P. Nettles C. D. Koester S. (2012). Attitudes toward methamphetamine use and HIV risk behavior in men who have sex with men. *Am. J. Addict.* 21 S35–S42. 10.1111/j.1521-0391.2012.00294.x 23786508

[B4] Beurmanjer H. Kamal R. M. de Jong C. A. J. Dijkstra B. A. G. Schellekens A. F. A. (2018). Baclofen to prevent relapse in gamma-hydroxybutyrate (GHB)-dependent patients: a multicentre, open-label, non-randomized, controlled trial. *CNS Drugs* 32 437–442. 10.1007/s40263-018-0516-6 29651711 PMC5976688

[B5] Bourne A. Reid D. Hickson F. Torres-Rueda S. Weatherburn P. (2015). Illicit drug use in sexual setting (« Chemsex ») and HIV/STI transmission risk behaviour among gay men in south London: findings from a qualitative study. *Sex. Trans. Infect.* 91 564–568. 10.1016/j.drugpo.2015.07.013 26163510

[B6] Bracchi M. Stuart D. Castles R. Khoo S. Back D. Boffito M. (2015). Increasing use of « party drugs » in people living with HIV on antiretrovirals: a concern for patient safety. *AIDS* 29 1585–1592. 10.1097/QAD.0000000000000786 26372268

[B7] Bui H. Zablotska-Manos I. Hammoud M. Jin F. Lea T. Bourne A. (2018). Prevalence and correlates of recent injecting drug use among gay and bisexual men in Australia: results from the FLUX study. *Int. J. Drug Pol.* 55 222–230. 10.1016/j.drugpo.2018.01.018 29429864

[B8] Busardo F. P. Gottardi M. Pacifici R. Vari M. R. Tini A. Volpe A. R. (2019). Nails analysis for drugs used in the context of chemsex: a pilot study. *J. Anal. Toxicol.* 10.1093/jat/bkz009 [Epub ahead of print]. 30855673

[B9] Busardo F. P. Gottardi M. Tini A. Minutillo A. Sirignano A. Marinelli E. (2018). Replacing GHB with GBL in recreational setting: a new trend in Chemsex. *Curr. Drug Metab* 19 1080–1085. 10.2174/1389200219666180925090834 30251602

[B10] Busardo F. P. Kyriakou C. Napoletano S. Marinelli E. Zaami S. (2015). Mephedrone related fatalities: a review. *Eur. Rev. Med. Pharmacol. Sci.* 19 3777–3790. 26502870

[B11] Cappetta M. Murnion B. P. (2019). Inpatient management of gamma-hydroxybutyrate withdrawal. *Austr. Psychiatry.* 27 284–287. 10.1177/1039856218822748 30652947

[B12] Chan P. A. Towey C. Poceta J. Rose J. Bertrand T. Kantor R. (2016). Online Hookup sites for meeting sexual partners among men who have sex with men in Rhode Island, 2013: a call for public health action. *Publ. Health Rep.* 131 264–271. 10.1177/003335491613100210 26957661 PMC4765975

[B13] Coppola M. Mondola R. (2012). Synthetic cathinones: chemistry, pharmacology and toxicology of a new class of designer drugs of abuse marketed as « bath salts » or « plant food ». *Toxicol. Lett.* 211 144–149. 10.1016/j.toxlet.2012.03.009 22459606

[B14] Corazza O. Assi S. Schifano F. (2013). From “Special K” to “Special M”: the evolution of the recreational use of ketamine and methoxetamine. *CNS Neurosci. Ther.* 19 454–460. 10.1111/cns.12063 23421859 PMC6493581

[B15] Dargan P. I. Sedefov R. Gallegos A. Wood D. M. (2011). The pharmacology and toxicology of the synthetic cathinone mephedrone (4-methylmethcathinone). *Drug. Test. Anal.* 3 454–463. 10.1002/dta.312 21755604

[B16] Degenhardt L. Mathers B. Guarinieri M. Panda S. Phillips B. Strathdee S. A. (2010). Reference group to the United Nations on HIV and injecting drug use. Meth/amphetamine use and associated HIV: implications for global policy and public health. *Int. J. Drug Pol.* 21 347–358. 10.1016/j.drugpo.2009.11.007 20117923

[B17] Diestelmann M. Zangl A. Herrle I. Koch E. Graw M. Paul L. D. (2018). MDPV in forensic routine cases: psychotic and aggressive behavior in relation to plasma concentrations. *Forensic Sci. Int.* 283 72–84. 10.1016/j.forsciint.2017.12.003 29275216

[B18] Dubov A. Galbo P. Altice F. L. Fraenkel L. (2018). Stigma and shame experiences by MSM who take PrEP for HIV prevention: a qualitative study. *Am. J. Mens Health.* 12 1843–1854. 10.1177/1557988318797437 30160195 PMC6199453

[B19] Edmundson C. Heinsbroek E. Glass R. Hope V. Mohammed H. White M. (2018). Sexualised drug use in the United Kingdom (UK): a review of the literature. *Int. J. Drug Pol.* 55 131–148. 10.1016/j.drugpo.2018.02.002 29625796

[B20] Elliot E. R. Singh S. Tyebally S. Gedela K. Nelson M. (2017). Recreational drug use and chemsex among HIV-infected in-patients: a unique screening opportunity. *HIV Med.* 18 525–531. 10.1111/hiv.12487 28117545

[B21] Ezaki J. Ro A. Hasegawa M. Kibayashi K. (2016). Fatal overdose from synthetic cannabinoids and cathinones in Japan: demographics and autopsy findings. *Am. J. Drug Alcohol Abuse* 42 520–529. 10.3109/00952990.2016.1172594 27283516

[B22] Fisher D. G. Reynolds G. L. Ware M. R. Napper L. E. (2011). Methamphetamine and Viagra use: relationship to sexual risk behaviors. *Arch. Sex. Behav.* 40 273–279. 10.1007/s10508-009-9495-5 19330436 PMC3047702

[B23] Forsyth A. J. (2012). Virtually a drug scare: mephedrone and the impact of the internet on drug news transmission. *Int. J. Drug Pol.* 23 198–209. 10.1016/j.drugpo.2011.12.003 22342603

[B24] Froberg B. A. Levine M. Beuhler M. C. Judge B. S. Moore P. W. Engebretsen K. M. (2015). Acute methylenedioxypyrovalerone toxicity. *J. Med. Toxicol.* 11 185–194. 10.1007/s13181-014-0446-8 25468313 PMC4469722

[B25] Garofalo R. Herrick A. Mustanski B. S. Donenberg G. R. (2007). Tip of the iceberg: young men who have sex with men, the Internet, and HIV risk. *Am. J. Public Health* 97 1113–1117. 10.2105/ajph.2005.075630 17463378 PMC1874202

[B26] Gilbart V. L. Simms I. Jenkins C. Furegato M. Gobin M. Oliver I. (2015). Sex, drugs and smart phone applications: findings from semistructured interviews with men who have sex with men diagnosed with *Shigella* flexneri 3a in England and Wales. *Sex Transm. Infect.* 91 598–602. 10.1136/sextrans-2015-052014 25921020

[B27] Giorgetti R. Tagliabracci A. Schifano F. Zaami S. Marinelli E. Busardo F. P. (2017). When « Chems » meet sex: a rising phenomenon called « ChemSex ». *Curr. Neuropharmacol.* 15 762–770. 10.2174/1570159X15666161117151148 27855594 PMC5771052

[B28] Grov C. Breslow A. S. Newcomb M. E. Rosenberger J. G. Bauermeister J. A. (2014). Gay and bisexual men’s use of the internet: research from the 1990s throught. *J. Sex Res.* 51 390–409. 10.1080/00224499.2013.871626 24754360 PMC4154140

[B29] Hammoud M. A. Vaccher S. Jin F. Bourne A. Haire B. Maher L. (2018). The new MTV generation: using metamphetamines. TruvadaTM, and ViagraTM to enhance sex and stay safe. *Int. J. Drug Pol.* 55 197–204. 10.1016/j.drugpo.2018.02.021 29526546

[B30] Heinsbroek E. Glass R. Edmundson C. Hope V. Desai M. (2018). Patterns of injecting and non-injecting drug use by sexual behaviour in people who inject drugs attending services in England, Wales and Northern Ireland, 2013-2016. *Int. J. Drug Pol.* 55 215–221. 10.1016/j.drugpo.2018.02.017 29523484

[B31] Hockenhull J. Murphy K. G. Paterson S. (2017). An observed rise in γ-hydroxybutyrate-associated deaths in London: evidence to suggest a possible link with concomitant rise in chemsex. *Forensic Sci. Int.* 270 93–97. 10.1016/j.forsciint.2016.11.039 27936427

[B32] Kalix P. (1990). Pharmacological properties of the stimulant khat. *Pharmacol. Ther.* 48 397–416. 10.1016/0163-7258(90)90057-9 1982180

[B33] Knight R. Karamouzian M. Carson A. Edward J. Carrieri P. Shoveller J. (2019). Interventions to address substance use and sexual risk among gay, bisexual and other men who have sex with men who use amphetamines: a systematic review. *Drug Alcohol Depend* 194 410–429. 10.1016/j.drugalcdep.2018.09.023 30502543

[B34] Kohli M. Hickson F. Free C. Reid D. Weatherburn P. (2019). Cross-sectional analysis of chemsex drug use and gonorrhoea diagnosis among men who have sex with men in UK. *Sex Health* 10.1071/SH18159 [Epub ahead of print]. 30760386

[B35] Kronstrand R. Guerrieri D. Vikingsson S. Wohlfarth A. Green H. (2018). Fatal poisonings associated with new psychoactive substances. *Handb. Exp. Pharmacol.* 252 495–541. 10.1007/164_2018_110 30105471

[B36] Lawn W. Aldridge A. Xia R. Winstock A. R. (2019). Substances-linked sex in heterosexual, homosexual, and bisexual men and women: an online, cross-sectional « global drug survey » report. *J. Sex Med.* 16 721–732. 10.1016/j.jsxm.2019.02.018 30952548

[B37] Lewnard J. A. Berrang-Ford L. (2014). Internet-based partner selection and risk for unprotected anal intercourse in sexual encounters among men who have sex with men: a meta-analysis of observational studies. *Sex Transm. Infect.* 90 290–296. 10.1136/sextrans-2013-051332 24518249

[B38] Liao P. C. Chang H. M. Chen L. Y. (2018). Clinical management of gamma-hydroxybutyrate (GHB) withdrawal delirium with CIWA-Ar protocol. *J. Formos. Med. Assoc.* 117 1124–1127. 10.1016/j.jfma.2018.06.005 29933901

[B39] Liechti M. (2015). Novel psychoactive substances (designer drugs): overview and pharmacology of modulators of monoamine signaling. *Swiss Med. Wkly.* 145:w14043. 10.4414/smw.2015.14043 25588018

[B40] Lucassen M. Samra R. Iacovides I. Fleming T. Shepherd M. Stasiak K. (2018). How LGBT+ young people use the Internet in relation to their mental health and envisage the use of e-therapy: exploratory study. *JMIR Serious Games* 6:e11249. 10.2196/11249 30578194 PMC6320432

[B41] Maxwell S. Shahmanesh M. Gafos M. (2019). Chemsex behaviours among men who have sex with men: a systematic review of the literature. *Int. J. Drug Pol.* 63 74–89. 10.1016/j.drugpo.2018.11.014 30513473

[B42] McKetin R. Dean O. M. Turner A. Kelly P. J. Quinn B. Lubman D. I. (2019). A study protocol for the N-ICE trial: a randomized double-blind placebo controlled study of the safety and efficacy of N-acetyl-cysteine (NAC) as a pharmacotherapy for methamphetamine (“ice”) dependence. *Trials* 20:325. 10.1186/s13063-019-3450-0 31164169 PMC6549263

[B43] McKetin R. Lunman D. I. Baker A. L. Dawe S. Ali R. L. (2013). Dose-related psychotic symptoms in chronic methamphetamine users: evidence from a prospective longitudinal study. *JAMA Psychiatry* 70 319–324. 10.1001/jamapsychiatry.2013.283 23303471

[B44] Medina M. M. Crowley C. Montgomery M. C. Tributino A. Almonte A. Sowemimo-Coker G. (2019). Disclosure of HIV serostatus and pre-exposure prophylaxis use on internet hookup sites among men who have sex with men. *AIDS Behav.* 23 1681–1688. 10.1007/s10461-018-2286-z 30267365 PMC6438768

[B45] Melendez-Torres G. J. Bourne A. Hickson F. Reid D. Weatherburn P. (2018a). Correlates and subgroups of injecting drug use in UK gay and bisexual men: findings from the 2014 gay men’s sex survey. *Drug Alcohol Depend.* 187 292–295. 10.1016/j.drugalcdep.2018.03.014 29702337

[B46] Melendez-Torres G. J. Bourne A. Reid D. Hickson F. Bonell C. Weatherburn P. (2018b). Typology of drug use in United Kingdom men who have sex with men and associations with socio-sexual characteristics. *Int. J. Drug Pol.* 55 159–164. 10.1016/j.drugpo.2018.01.007 29398203

[B47] Morgan C. J. Curran H. V. (2012). Ketamine use: a review. *Addiction* 107 27–38. 10.1111/j.1360-0443.2011.03576.x 21777321

[B48] Neeki M. N. Dong F. Liang L. Toy J. Carrico B. Jabourian N. (2018). Evaluation of the effect of methamphetamine on traumatic injury complications and outcomes. *Addict Sci. Clin. Pract.* 13 11. 10.1186/s13722-018-0112-6 29592800 PMC5874991

[B49] Neu P. (2019). Course and complications of GHB detoxification treatment a 1-year case series. *Nervenarzt* 90 509–515. 10.1007/s00115-018-0636-8 30362026

[B50] O’Connor A. D. Padilla-Jones A. Gerkin R. D. Levine M. (2015). Prevalence of rhabdomyolysis in sympathomimetic toxicity: a comparaison of stimulants. *J. Med. Toxicol.* 11 195–200. 10.1007/s13181-014-0451-y 25468315 PMC4469713

[B51] Papaseit E. Olesti E. de la Torre R. Torrens M. Farre M. (2017). Mephedrone concentrations in cases of clinical intoxication. *Curr. Pharm. Des.* 23 5511–5522. 10.2174/1381612823666170704130213 28677506

[B52] Parson J. T. John S. A. Millar B. M. Starcks T. J. (2018). Testing the efficacy of combined motivation interview and cognitive behavioral skills training to reduce methamphetamine use and improve HIV medication adherence among HIV-positive gay and bisexual men. *AIDS Behav.* 22 2674–2686. 10.1007/s10461-018-2086-5 29536284 PMC6051905

[B53] Penders T. M. Gestring R. E. Vilensky D. A. (2012). Excited delirium following use of synthetic cathinones (bath salts). *Gen. Hosp. Psychiatry* 34 647–650. 10.1016/j.genhosppsych.2012.06.005 22898445

[B54] Petit A. Karila L. Sananes M. Lejoyeux M. (2013). Mephedrone: a new synthetic drug. *Press Med.* 42 1310–1316. 10.1016/j.lpm.2012.11.019 23669315

[B55] Rana S. Macdonald N. French P. Jarman J. Patel S. Portman M. (2019). Enhanced surveillance of syphilis cases among men who have sex with men in London, October 2016-January 2017. *Int. J. Std. Aids* 30 422–429. 10.1177/0956462418814998 30621550

[B56] Raposo Pereira F. McMaster M. T. B. de Vries Y. D. A. T. Polderman N. van den Brink W. van Wingen G. A. (2019b). Influence of gamma-hydroxybutyric acid-use and gamma-hydroxybutyric acid-induced coma on affect and the affective network. *Eur. Addict. Res.* 25 173–181. 10.1159/000497381 30999293 PMC7050670

[B57] Raposo Pereira F. McMaster M. T. B. Polderman N. de Vries Y. D. A. T. van den Brink W. van Wingen G. A. (2018). Adverse effects of GHB-induced coma on long-term memory and realted brain function. *Drug Alcohol Depend* 190 29–36. 10.1016/j.drugalcdep.2018.05.019 29966850

[B58] Raposo Pereira F. McMaster M. T. B. Schellekens A. Polderman N. de Vries Y. D. A. T. van den Brink W. (2020). Effects of recreational GHB and multiple GHB-induced comas on brain structure and impulsivity. *Front. Psychiatry.* 11:166. 10.3389/fpsyt.2020.00166 32300311 PMC7142256

[B59] Raposo Pereira F. Zhutovsky P. Mcmaster M. T. B. Polderman N. de Vries Y. D. A. T. van den Brink W. (2019a). Recreational use of GHB is associated with alterations of resting state functional connectivity of the central executive and default mode networks. *Hum. Brain Mapp.* 40 2413–2421. 10.1002/hbm.24532 30720906 PMC6590661

[B60] Reback C. J. Shoptaw S. (2014). Development of an evidence-based, gay-specific cognitive behavioral therapy intervention for methamphetamine-abusing gay and bisexual men. *Addict. Behav.* 39 1286–1291. 10.1016/j.addbeh.2011.11.029 22169619 PMC3326187

[B61] Renninger N. J. (2018). Grindr killed the gay bar, and other attemps to blame social technologies for urban development: a democratic approach to popular technologies and queer sociality. *J. Homosex.* 17 1–20. 10.1080/00918369.2018.1514205 30222066

[B62] Riley A. L. Nelson K. H. To P. Lopez-Arnau R. Xu P. Wang D. (2019). Abuse potential and toxicity of the synthetic cathinones (i.e*., « Bath salts »)*. *Neurosci. Biobehav. Rev.* 10.1016/j.neurobiorev.2018.07.015 [Epub ahead of print]. PMC8617600 31101438

[B63] Rommel N. Rohleder N. H. Koerdt S. Wagenpfeil S. Härtel-Petri R. Wolf K. D. (2016). Sympathomimetic effects of chronic methamphetamine abuse on oral health: a cross-sectional study. *BMC Oral Health* 16:59. 10.1186/s12903-016-0218-8 27388625 PMC4937577

[B64] Sande M. (2016). Characteristics of the use of 3-MMC and other new psychoactive drugs in Slovenia, and the perceived problems experienced by users. *Int. J. Drug Pol.* 27 65–73. 10.1016/j.drugpo.2015.03.005 25908121

[B65] Schmidt A. J. Bourne A. Weatherburn P. Reid D. Marcus U. Hickson F. (2016). Illicit drug use among gay and bisexual men in 44 cities: findings from the European MSM internet survey (EMIS). *Int. J. Drug Pol.* 38 4–12. 10.1016/j.drugpo.2016.09.007 27788450

[B66] Schmoll S. Romanek K. Stich R. Bekka R. Stenzl J. Geith S. (2018). An internet-based survey of 96 german-speaking users of “bath salts”: frequent complications, risky sexual behavior, violence, and delinquency. *Clin. Toxicol.* 56 219–222. 10.1080/15563650.2017.1353094 28753045

[B67] Scott J. C. Woods S. P. Matt G. E. Meyer R. A. Heaton R. K. Atkinson J. H. (2007). Neurocognitive effects of methamphetamines: a critical review and meta-analysis. *Neuropsychol. Rev.* 17 275–297. 10.1007/s11065-007-9031-0 17694436

[B68] Serre A. Vuillot O. Eiden C. Gambier J. Berger A. Mathieu O. (2019). Acute Psychiatric Disorders Related to fake Cathinones: Ephylone. *J. Anal. Toxicol.* 43 e1–e2. 10.1093/jat/bkz020 31095710

[B69] Sevak R. J. Vansickel A. R. Stoops W. W. Glaser P. E. A. Hays L. R. Rush C. R. (2011). Discriminative-stimulus, subject-rated, and physiological effects of methamphetamine in human pretreated with aripiprazole. *J Clin. Psychopharmacol.* 31 470–480. 10.1097/JCP.0b013e318221b2db 21694622 PMC3712353

[B70] Sewell J. Cambiano V. Miltz A. Speakman A. Lampe F. C. Philipes A. (2018). Changes in recreational drug use, drug use associated with chemsex, and HIV-related behaviours, among HIV-negative men who have sex with men in London and Brighton, 2013-2016. *Sex Transm. Infect.* 94 494–501. 10.1136/sextrans-2017-053439 29700052 PMC6227813

[B71] Sewell J. Cambiano V. Speakman A. Lampe F. C. Phillips A. Stuart D. (2019). Changes in chemsex and sexual behaviour over time, among a cohort of MSM in London and Brighton: findings from the AURAH2 study. *Int. J. Drug Policy.* 68 54–61. 10.1016/j.drugpo.2019.03.021 30999243

[B72] Shetty V. Harrell L. Murphy D. A. Vitero S. Gutierrez A. Belin T. R. (2015). Dental disease patterns in methamphetamine users: findings in a large urban sample. *J. Am. Dent. Assoc.* 146 875–885. 26610832 10.1016/j.adaj.2015.09.012 PMC5364727

[B73] Soriano V. Romero J. D. (2018). Rebound in sexually transmitted infections following the success of antiretrovirals for HIV/AIDS. *AIDS Rev.* 20 187–204. 10.24875/AIDSRev.18000034 30548023

[B74] Spiller H. A. Ryan M. L. Weston R. G. Jansen J. (2011). Clinical experience with and analytical confirmation of “bath salts” and “legal highs” (synthetic cathinones) in the United States. *Clin. Toxicol.* 49 499–505. 10.3109/15563650.2011.590812 21824061

[B75] Spindler H. H. Scheer S. Chen S. Y. Klausner J. D. Katz M. H. Valleroy L. A. (2007). Viagra, methamphetamine, and HIV risk: results from a probability sample of MSM. *San Francisco*. *Sex Transm Dis.* 34 586–591. 10.1097/01.olq.0000258339.17325.93 17334264

[B76] Tang W. Best J. Zhang Y. Liu F. Y. Tso L. S. Huang S. (2016). Gay mobile apps and the evolving virtual risk environment: a cross-sectional online survey among men who have sex with men in China. *Sex Transm. Infect.* 92 508–514. 10.1136/sextrans-2015-052469 27288414 PMC5148710

[B77] Umebachi R. Aoki H. Sugita M. Taira T. Wakai S. Saito T. (2016). Clinical characteristics of α-pyrrolidinovalerophenone (α-PVP) poisoning. *Clin. Toxicol.* 54 563–567. 10.3109/15563650.2016.1166508 27227375

[B78] Vaux S. Chevaliez S. Saboni L. Sauvage C. Sommen C. Barin F. (2019). Prevalence of hepatitis C infection, screening and associated factors among men who have sex with men attending gay venues: a cross-sectional survey (PREVAGAY), France 2015. *BMC Infect. Dis.* 19:315. 10.1186/s12879-019-3945-z 30971207 PMC6458747

[B79] Whitfield D. L. Kattari S. K. Walls N. E. Al-Tayyib A. (2017). Grindr, Scruff, and on the hunt: predictors of condomless anal sex, Internet use, and mobile application use among men who have sex with men. *Am. J. Mens. Health* 11 775–784. 10.1177/1557988316687843 28134002 PMC5675229

[B80] Winstock A. Mitcheson L. Ramsey J. Davies S. Puchnarewicz M. Marsden J. (2011). Mephedrone: use, subjective effects and health risks. *Addiction* 106 1991–1996. 10.1111/j.1360-0443.2011.03502.x 21592252

[B81] Wood D. M. Greene S. L. Dargan P. I. (2013). Five-year trends in self-reported recreational drugs associated with presentation to a UK emergency department with suspected drug-related toxicity. *Eur. J. Emerg. Med.* 20 263–267. 10.1097/MEJ.0b013e3283573115 22850087

